# Editorial: Origins of Human Neuropathology: The Significance of Teneurin-Latrophilin Interaction

**DOI:** 10.3389/fnins.2020.00501

**Published:** 2020-05-15

**Authors:** David A. Lovejoy, Antony A. Boucard, Richard P. Tucker

**Affiliations:** ^1^Department of Cell and Systems Biology, University of Toronto, Toronto, ON, Canada; ^2^Departamento de Biología Celular, Centro de Investigación y de Estudios Avanzados del Instituto Politécnico Nacional, Mexico, Mexico; ^3^Department of Cell Biology and Human Anatomy, University of California, Davis, Davis, CA, United States

**Keywords:** G-protein couple receptor, adhesion, affective disorders, molecular evolution, transmembrane proteins, neural development, neuroplasticity, synapse formation

We are delighted to introduce this new special issue on “The Origins of Neuropathology: The Significance of Teneurin-Latrophilin Interaction.” Although the title may seem particularly bold, and indeed, perhaps presumptuous, we the editors, think our title well warranted based on the findings and interpretation provided by a dedicated group of researchers who have developed this field over the last 25 years. In this publication, we introduce the readers to researchers whom have pioneered this field, and those whom have played an essential role in developing this research direction. Now, together, their combined work have elucidated a novel ligand-receptor network that evolved during the earliest period of animal evolution, and has fostered a new insight into the ancient evolutionary organization of the central nervous system (CNS). Specifically, this work offers a new understanding of several aspects of neuropathology including degenerative, psychiatric and mood disorders and, furthermore, illuminates a fundamental role that teneurins and latrophilins play in cell-to-cell metabolism that may be associated with various forms of cancer both within and outside of the brain.

In 1994, the laboratories of Professors Ron Wides in Israel and Ruth Chiquet-Ehrismann working in Switzerland, independently reported the existence of a novel transmembrane protein and its gene in *Drosophila*. A complex gene/protein, its closest homolog was that of the tenascins. The gene was named either odd oz (odz) or tenascin major (ten-m) by these researchers. Subsequent studies indicated that the gene was highly expressed in the brains of vertebrates and the term “teneurin” was coined to reflect both its relationship with tenascins and with the CNS. Around the same time as these studies, a novel G protein-coupled receptor was identified by Ushkaryov et al. in the United Kingdom (in fact the latrophilins then named CIRL, calcium-independent receptor for α-latrotoxin, was first identified by the group of Petrenko at NYU Medical Center in New York, USA), which was subsequently established as a cognate receptor for the teneurins. This receptor was later termed as the latrophilins and more recently “Adhesion receptor G-protein coupled receptor, family L, or ADGRL”.

In Part 1 of this publication, the early history on the origin and discovery of teneurins has been described by Baumgartner and Wides, Wides, and Tucker. Recent structural studies by Jackson et al., as well as Araç and Li have provided molecular models to understand how teneurins are ensconced in the plasma membrane and play a role in synaptic interaction. In addition, their work integrates the molecular mechanisms with the early evolution of both teneurins and latrophilins.

In Part 2, four studies build upon the evolutionary development of teneurins by examining its role in nematodes by Topf and Drabikowski, a model of teneurin action in the *Drosophilia* nervous system by DePew et al.; and two studies on fish. Angela Cheung et al. describe the neurological function and expression in zebrafish, whereas Reid et al. have described novel actions of the teneurins with respect to metabolism in fish.

Part 3 of this publication is focused on the latrophilins and is led off by Ushkaryov et al. describing the discovery, structure, and function of the latrophilins. This work is followed by a review by Moreno-Salinas et al. in Antony Boucard's laboratory describing the structure of the latrophilins and its interaction with associated transmembrane proteins with respect to adhesion, neuronal function, and pathology. The following paper, by Schöneberg and Prömel links the previous papers with a comparison of teneurin and latrophilin interactions in invertebrates and vertebrates. Finally, in this section, Burbach and Meijer provide an interesting overview of the relationship of teneurins and latrophilins with respect to other proteins described in these other papers. Together, these studies provide a novel understanding of how the teneurins and latrophilins interact in a complex set of associated proteins.

The next section (Part 4) of the publication focuses on the development and maintenance of the CNS in mammals. Here, Leamey and Sawatari lead off with a discussion of the role of teneurin-associated neuro-circuit formation using knockout studies in mice. A detailed review by Sita et al. in the Bittencourt laboratory frames this and previous studies in a comparative neuroanatomical background, and in addition, provides a neuroanatomical rationale for new studies associated with other regions of the CNS. Building upon these studies, Hogg et al. include a review on the behavioral actions of the teneurin C-terminal associated peptide (TCAP) in mammals and its potential relationship to brain metabolism and forms of neuropathology. Finally, in this section, a study by Tessarin et al. in the Casatti laboratory shows for the first time, teneurins may be associated with astrocyte function, indicating a novel function for teneurins with respect to some glial-based disorders in the brain.

Finally in our last section, we have provided some studies on the potential roles of the teneurins and latrophilins with respect to carcinogenesis. Although these studies are somewhat removed from our treatise on the role of teneurins and latrophilins with respect to neuronal development, maintenance and pathology, they provide interesting observations that may be relevant to some types of CNS pathology. Thus, Rebolledo-Jaramillo and Ziegler include a review on the relationship of teneurins to several types of cancers. This is followed by a research report by Husić et al. suggesting that the TCAP region of the teneurins could play a role in modulating the adhesion of the cancer-like cell line, HEK293 and finally, Bastias-Candia et al. and associates have provided novel data on the role of teneurin-3 with respect to Wnt signaling and have discussed its potential role in neural development and carcinogenesis.

Overall, we posit that the teneurins and latrophilins played a major role in the early evolution of the nervous system and may underlie the etiology of a number of neurological disorders that are thus-far misunderstood. Indeed, we hope that this publication will stimulate further research into the actions of teneurins and latrophilins and lead to novel approaches of understanding and ultimately treatment.

## Ruth Chiquet-Ehrismann (1954-2015): A Teneurin Pioneer

A major player in the discovery and characterization of teneurins was the Swiss scientist, Ruth Chiquet-Ehrismann. Dr. Chiquet-Ehrismann had a long-standing interest in cell-cell and cell-extracellular matrix interactions, particularly during development and tumorigenesis. She earned her Ph.D. at the ETH Zurich under the mentorship of David C. Turner, where she performed early work on the cell and heparin-binding sites of fibronectin. Shortly after joining the Friedrich Miescher Institute in Basel as a junior group leader in 1984, Ruth, in collaboration with Eleanor J. Mackie and Teruyo Sakakura, published a paper in *Cell* describing an extracellular matrix glycoprotein that she named “tenascin.” A key observation made in this widely cited paper was the presence of tenascin in the extracellular matrix of embryonic tissues and the stroma of breast cancer, but its absence from most normal adult tissues. We now know that the original “tenascin” was the founding member of a diverse gene family, and that members of this family promote cell motility, proliferation, and differentiation in a variety of tissue environments, both normal and pathological.

But in the early 1990s, it was unclear how tenascins functioned. Specifically, its receptors and binding partners were not understood. Subsequently, Ruth engaged in a multi-pronged approach to studying tenascin function in an attempt to identify its homologs in *Drosophila*. This work, led by her postdoctoral fellow Dr. Stefan Baumgartner, resulted in the discovery of a novel family of type-2 transmembrane proteins that they named ten-a and ten-m, for “tenascin-like proteins accessory and major.” When the homologs of ten-a and ten-m were found in vertebrates and they were shown to be highly expressed in the nervous system, Ruth proposed the name “teneurins.” This name combined the names of the original proteins from *Drosophila* with neurons, which appeared to be their most prominent site of expression.

From that point onward, Ruth's research group at the Friedrich Miescher Institute studied two topics: the roles of tenascins in cancer and the roles of teneurins in development. Using numerous model systems, her research included studies of teneurins in arthropods (*Drosophila)*, nematodes (*C. elegans)* and chordates (birds and humans). Key firsts that came from Ruth's laboratory include the cloning and sequencing of human teneurins, experimental evidence of teneurin processing by furin and the potential nuclear localization of the intracellular domain, the ability of teneurins to promote growth cone spreading, patterning defects in teneurin knockout animals, a description of the ancient origins of teneurins via horizontal gene transfer, the complementary expression patterns of different teneurins during development, the cytotoxic properties of the teneurin C-terminal domain, and the presence of homotypic adhesion domains in teneurins.

Since 1994, Ruth's group published 24 papers on the cloning, expression, origins and functions of teneurins. Contributing to these papers were 15 graduate students and postdoctoral fellows, often with the expert technical guidance of Jacqueline Ferralli, Marianne Brown-Luedi, and Doris Martin. This work has provided a foundation for a new generation of researchers in the field of teneurins.

Ruth Chiquet-Ehrismann passed away at her home near Basel on September 4, 2015. She is survived by her husband and collaborator Matthias Chiquet, three children, Daniel, Patrice and Fabian, and an expanding cohort of grandchildren.

## Author Contributions

All authors listed have made a substantial, direct and intellectual contribution to the work, and approved it for publication.

## Conflict of Interest

The authors declare that the research was conducted in the absence of any commercial or financial relationships that could be construed as a potential conflict of interest.

